# Brothers in arms: emerging roles of RNA epigenetics in DNA damage repair

**DOI:** 10.1186/s13578-017-0151-9

**Published:** 2017-05-04

**Authors:** Jinwei Zhang

**Affiliations:** Laboratory of Molecular Biology, National Institute of Diabetes and Digestive and Kidney Diseases, 50 South Drive, Room 4503, Bethesda, MD 20892 USA

## Abstract

N6-methyladenosine (m^6^A) is a widespread posttranscriptional RNA modification that occurs in tRNA, rRNA, snRNA, viral RNAs, and more recently is shown to occur in mRNA in a dynamic, reversible manner. At the epicenter of RNA epigenetics, m^6^A influences essentially all stages of RNA metabolism. As a result, m^6^A modulates cell differentiation and pluripotency, cell cycle and tumorigenesis, and several types of stress responses, etc. A recent report by Shi and colleagues uncovers a novel pathway in which m^6^A RNA, its associated enzymes, and DNA polymerase κ constitute an early-response system that confers cellular resistance to ultraviolet irradiation, separate from the canonical nucleotide excision repair (NER) pathway that normally repairs UV-induced DNA damage.

## Background

**Fig. 1:**
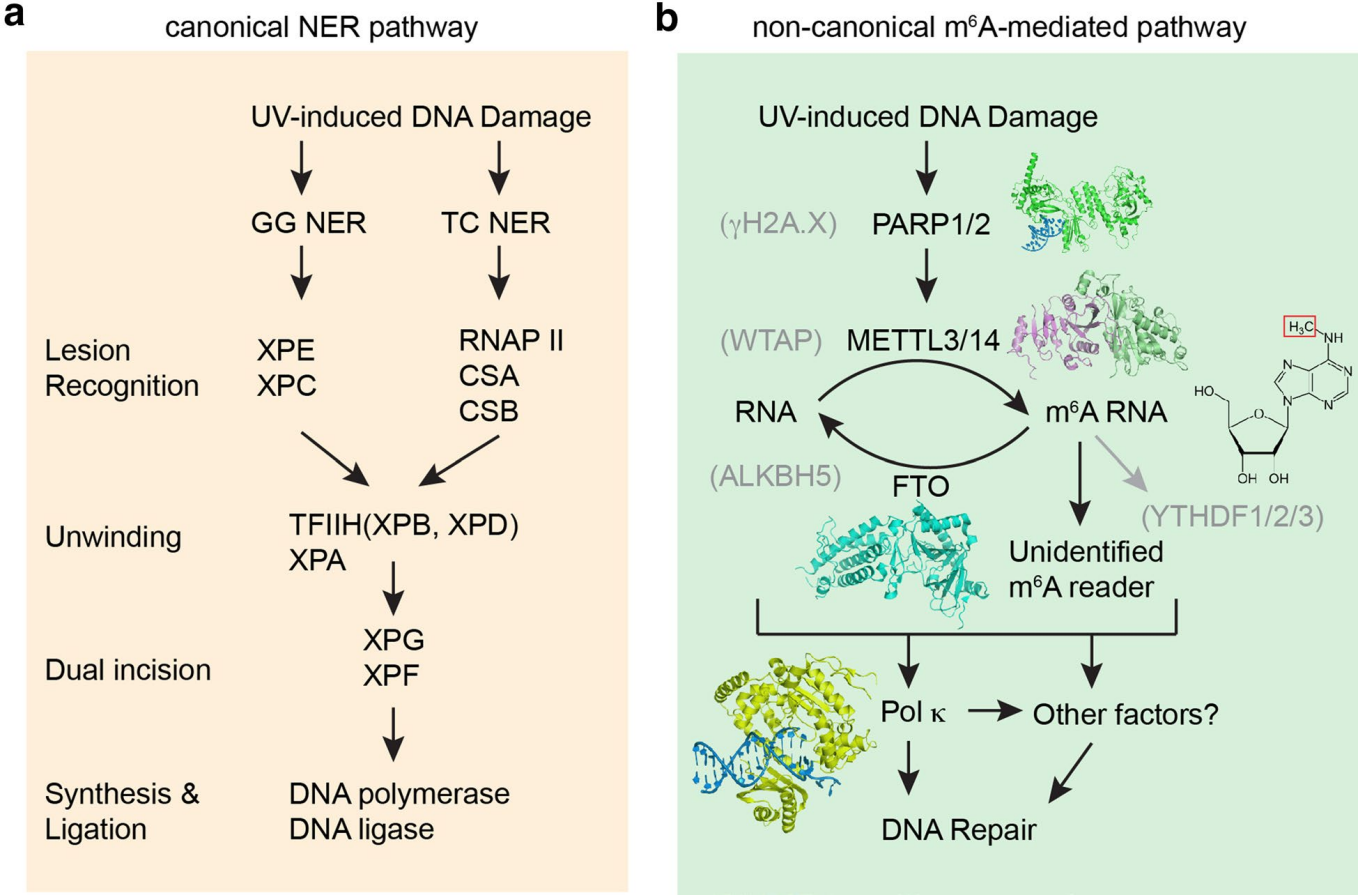
The canonical nucleotide excision repair (NER) pathway (**a**) and the novel m^6^A RNA-mediated DNA damage repair pathway (**b**). **a** Steps and components of the NER pathway [10]. GG (global genomic) NER and TC (transcription coupled) NER differ in the initial recognition of the DNA lesions but subsequently converge into the same pathway that unwinds, excises, and replaces the damaged DNA segment. **b** Steps and known components of the new, m^6^A-mediated pathway. Factors indicated in* gray* are shown to be not contributing to this pathway [9]. Representative crystal structures of PARP (PDB: 4DQY), METTL3/14 (PDB: 5IL0), FTO (PDB: 3LFM), and Pol κ (PDB: 2W7O) are rendered with MacPyMOL

Among the trio of linear heteropolymers that enable organic life as we know it, RNAs are recognized as the most versatile and self-sufficient. In order to serve its primary directive in the faithful preservation and propagation of genetic information to subsequent generations, DNA assumes a protective, inert duplex structure where sequence information is carefully concealed in is hydrophobic, stacked central axes. Further, the genetic information is maintained in duplicates, in the form of two paired strands, each replete with the 4-bit informational strings. Despite such extraordinary measures to preserve the genetic information, cellular DNA is subjected to a constant onslaught of chemical and environmental insults that create lesions that interfere with its replication, transcription, modification, etc. Depending on the source (ROS, UV, X-rays, Gamma rays, toxins, mutagens, etc.) and type of DNA damage (oxidation, alkylation, hydrolysis, adducts, mismatches, etc.), cells employ a battery of DNA repair pathways to detect and correct the damages. These include base excision repair (BER), nucleotide excision repair (NER; Fig. 1), mismatch repair, etc. that act on single-strand damages, as well as non-homologous end joining (NHEJ), microhomology-mediated end joining (MMEJ), and homologous recombination (HR), which repair double-strand breaks (DSBs) [1].

In contrast to DNA, RNA, or “the other nucleic acid”, is unburdened from the task of genetic information preservation and thus liberated from the mundane duplex structure of DNA. Instead, it is free to assume single- and double-stranded structures and can fold into tertiary and quaternary structures that rival large proteins in architectural sophistication and complexity. This is exemplified by some of the most important extant cellular machineries such as the ribosome, spliceosome, telomerase, RNase P and other ribozymes, and riboswitches, etc. Such structural versatility, combined with its short-lived nature, allow RNA to exert an increasingly sophisticated panel of non-inherited, regulatory functions outside of its template role in translation. However, compared to the protein polymers that have gradually taken over most RNA functions since the primordial RNA world, RNA suffers a debilitating lack of chemical diversity on its side chains (4 versus 20). To remedy such limitations, RNA has evolved more than 100 types of chemical elaborations on its nucleobase and ribose moieties. For instance, an average 12% of nucleotides on tRNA (~8 per tRNA molecule) are post-transcriptionally modified. RNA posttranscriptional modifications confer numerous advantages and nuances in modulating RNA structure and function, some of which are further dynamically coupled with the metabolic state of the cell.

### A thriving renaissance of RNA epigenetics

Using genome-wide RNA sequencing methods that can detect and enrich specific modified RNA, it was recently discovered that in addition to tRNAs, rRNAs, snRNAs, etc., that are canonically modified, mRNAs are also elaborated with a number of distinct chemical modifications including N6-methyladenosine (m^6^A), N1-methyladenosine (m^1^A), pseudouridine (ψ), 5-methylcytosine (m^5^C), and 2′-*O*-methylation (2′-OMe), etc. [2]. These discoveries have ushered in a burgeoning renaissance of RNA modification research, or RNA epigenetics. Among these, m^6^A, or N6-methyladenosine modification, is the most abundant internal modification in eukaryotic mRNA (~3–5 sites per mRNA) [3, 4]. m^6^A plays wide-ranging roles in essentially all known steps of RNA metabolism, affecting RNA splicing and processing, nuclear export and localization, translation and stability, etc. These effects are manifest in phenotypic effects on cell differentiation, pluripotency, tumorigenesis, circadian rhythm, etc. [2, 5]. Further, m^6^A is involved in several types of stress responses, including heat shock, nitrogen starvation, oxidative stress, hypoxia, etc. [6, 7]. The deposition of m^6^A modification can lead to immediate remodeling of RNA secondary and tertiary structures, as m^6^A weakens duplex formation and stabilizes stacking within single-stranded regions. Such thermodynamic effects not only modulate RNA structure, but also regulate its access by RNA-binding proteins such as HNRNPC, constituting “m^6^A switches” [8]. Taken together, the dynamic, reversible m^6^A modification is well suited for fast-acting responses to cellular and environmental stimuli.

Along the emerging theme of m^6^A acting as early responders of stress response, Shi, He, and colleagues have recently reported a remarkable discovery that m^6^A in RNA, its associated enzymes Poly-ADP ribose polymerase (PARP), N6-adenosine-methyltransferase heterodimer (METTL3/14), Fat mass and obesity-associated protein (FTO), and DNA polymerase κ (Pol κ), form a novel, dynamic pathway that launches a rapid response (within 2 min) to UV-induced DNA damage [9].

### A novel RNA-mediated DNA repair pathway

Using laser micro-irradiation or global UVC irradiation of U2OS cells and an antibody against m^6^A, Xiang et al., detected rapid, reversible accumulation of m^6^A RNA at the sites of irradiation [9]. The authors identified the METTL3/METTL14 complex (but not METTL3/WTAP complex) and FTO (but not ALKBH5) as the writer and eraser of the m^6^A mark at these DNA damage sites (Fig. 1). The factor that operates upstream of the RNA methylation is the early DNA damage responder PARP but not γH2A.X. Interestingly, this m^6^A formation is specific for DNA damage induced by UV irradiation and is not triggered by infrared or γ irradiation or chemical insults [9].

Enzymatically active METTL3, the methylation writer rapidly recruited to the UV-irradiated DNA damage site, plays a central role in this novel pathway. METTL3 KO cells are characterized by the hindered removal of cyclobutane pyrimidine dimers (CPDs, primary lesions from UV exposure), an inability to recruit downstream effector Pol κ to the damage site, significantly delayed re-initiation of nascent transcription after DNA damage, and ultimately drastically reduced cell survival [9]. Remarkably, exogenous over-expression of Pol κ rescues the CPD removal defect, suggesting that rapid Pol κ recruitment to the damage site is potentially the purpose of m^6^A deposition. It remains unknown how exactly Pol κ is recruited to the damage site and whether its translesion synthesis activity is responsible for the UV resistance conferred by this m^6^A pathway. Importantly, this m^6^A-mediated DNA damage response is separate from the canonical NER and Rad18/PCNA pathways, which also recruit Pol κ to DNA damage sites, albeit at a much later time (10–30 min; Fig. 1).

## Conclusion and perspectives

This timely work expands the already impressive repertoire of cellular functions m^6^A plays and makes a surprising connection between m^6^A RNA biology and DNA repair. Like any other transformative discovery, this work raises more questions that it answers. For example, are specialized transcripts or all nearby RNAs responsible for aiding DNA damage repair? The authors address this question by capturing the GGACU motif used for METTL3 recruitment and demonstrate the enrichment of UV-induced m^6^A in 5′ untranslated regions (UTR) in a metagene analysis. This finding echoes an earlier analysis of the heat shock response, where increased m^6^A in 5′ UTR promotes cap-independent translation [7]. Are these marked RNA physically associated with chromatin and are they located near their site of transcription? Do RNA transcripts serve a general protective role for chromatin against damage? Could the m^6^A-marked transcripts play a more direct role besides recruiting METTL3? Besides known protein-binding primary sequence motifs, could the RNA secondary, tertiary, or quaternary structures (which are directly modulated by the deposition and removal of epigenetic marks) contribute to factor recruitment or other functions in this context? Which protein reads the UV-induced m^6^A deposition and is this protein or the RNA or both that ultimately recruit Pol κ? What is special about Pol κ that contributes to its unique recruitment and activity at the damage site, not possessed by other DNA polymerases such as Pol δ, ε, η, ι, etc.?

Nevertheless, what is clear is that this landmark paper leaves us with a fertile area for future research and makes one wonder what other pathways would be next to cross paths with RNA epigenetics and noncoding RNA biology.
